# Converging on the Origins of Axonal Ion Channel Clustering

**DOI:** 10.1371/journal.pgen.1000340

**Published:** 2009-01-16

**Authors:** Matthew N. Rasband

**Affiliations:** Department of Neuroscience, Baylor College of Medicine, Houston, Texas, United States of America; The Jackson Laboratory, United States of America

The high-density clustering of voltage-gated Na^+^ channels and KCNQ2/3 K^+^ channels at the axon initial segment (AIS; see Figure 1A) and nodes of Ranvier (Figure 1B) is essential for the final integration of synaptic inputs and the initiation and rapid propagation of action potentials (APs) in neurons [1]–[3]. The AIS also marks the transition between the axonal and somatodendritic domains of the neuron, and its molecular integrity is required to maintain neuronal polarity [4],[5]. Thus, the AIS acts as both a key functional and structural bridge between neuronal input and output. Previously, the cytoskeletal and scaffolding protein ankyrinG was shown to be essential for the clustering of Na^+^ channels at the AIS and at the peripheral nervous system nodes of Ranvier [6]–[8]. Efforts to identify the molecular basis of Na^+^ channel clustering at the AIS showed that all mammalian Na^+^ channels have a cytoplasmic anchor motif that mediates their interaction (and AIS clustering) with ankyrinG [9],[10]. Subsequently, a comparison between Na^+^ channels and KCNQ2/3 K^+^ channels revealed the surprising fact that these two types of channels have the ankyrinG interaction and anchor motif in common [11]. While these anchor motifs are highly conserved among vertebrates, they are not found among invertebrates. This observation led to the fascinating question of how and why ion channels from two different gene families evolved a common amino acid sequence that mediates their clustering and localization at the AIS and nodes of Ranvier.

**Figure 1:**
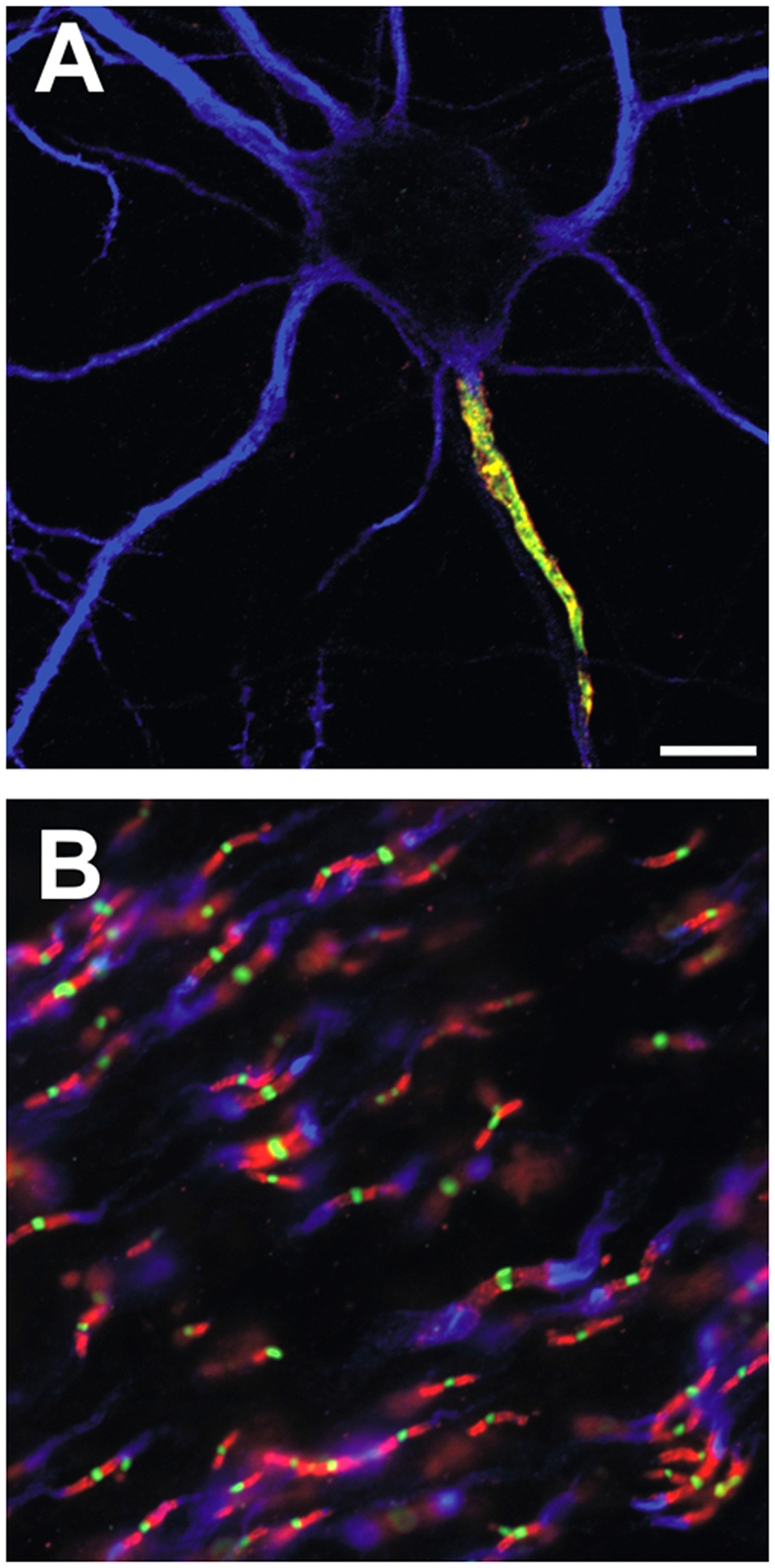
Ion channels are clustered in mammalian axons at the AIS and nodes of Ranvier. (A) A hippocampal neuron in culture is labeled for Na^+^ channels (red), the cytoskeletal and ankyrinG interacting protein βIV spectrin (green), and MAP2 (blue), to indicate the somatodendritic domain. (B) Nodes of Ranvier are triple-labeled for Nav channels (green), caspr to label paranodal junctions (red), and Kv1 K^+^ channels to label juxtaparanodal regions of myelinated axons.

In a remarkable follow-up paper published in a recent issue of *PLoS Genetics*, Hill et al. [12] have now addressed this question by identifying key steps in the evolution of ion channel clustering on axons. They performed a phylogenetic analysis of invertebrate and vertebrate Na^+^ channels to define where along the evolutionary tree the AIS targeting motifs arose. They found that the first Na^+^ channels with anchor motifs arose in basal chordates, such as amphioxus, and that all orthologous Na^+^ channel genes in jawed vertebrates contain this anchor motif. The anchor motif is encoded by a single exon that is not found among invertebrates. Hill et al. [12] then used Na^+^ channel antibodies directed against a highly conserved sequence in the inactivation gate to discern the distribution of Na^+^ channels in dye-filled motor neurons from lamprey, a primitive, jawless vertebrate lacking myelin. They observed a narrow region of the axon, adjacent to the neuronal cell body, that was highly enriched in Na^+^ channels and that resembled the corresponding AIS found in mammals (Figure 1A). Consistent with the idea that these channels underlie the AP in lamprey neurons, previous studies showed that lamprey motor neuron APs are initiated in the proximal axon [13]. Thus, Hill et al. [12] demonstrate that a morphologically, molecularly, and functionally distinct AIS arose in basal chordates before the evolution of myelin and nodes of Ranvier.

Although KCNQ2/3 K^+^ channel homologs could be identified in lamprey, in contrast to Na^+^ channels, they lacked an AIS anchor motif. Instead, Hill et al. [12] found that the KCNQ2/3 K^+^ channel AIS anchor motif arose after lampreys diverged from other vertebrates, in a common ancestor of shark and humans. This sequence of evolutionary events is approximately coincident with that for the evolution of myelin, suggesting that the unique properties of vertebrate myelinated axons (i.e., saltatory conduction) drove the molecular evolution of the KCNQ2/3 K^+^ channels so that they, too, localized at nodes and the AIS.

The results of Hill et al. [12] are both significant and profound. Their results provide a first glimpse into the evolutionary origins of ion channel clustering along axons. The phylogenetic analysis of the anchor motifs in Na^+^ and KCNQ2/3 K^+^ channels suggests that nodes of Ranvier evolved from the previously developed AIS. This view is consistent with the AIS being specified intrinsically and assembled by the neuron, but node formation is initiated by, and requires, extrinsic factors derived from myelinating glia, some of which have recently been described [14]. Thus, channel clustering at the AIS may have been a key evolutionary event, facilitating the subsequent development of myelin, saltatory conduction, and the complex vertebrate nervous system.

The analysis of Na^+^ and KCNQ2/3 K^+^ channel clustering mechanisms also provides a remarkable instance of convergent evolution. While many examples exist for functional and anatomical convergence as a consequence of different organisms occupying similar ecological niches, the work of Hill et al. [12] demonstrates that the AIS anchor motifs are analogous structures that arose through molecular convergence, implying that Na^+^ and KCNQ2/3 K^+^ channels occupy a similar molecular and functional niche. The restricted localization of these channels to the nodes and the AIS strongly supports this view. However, because ankyrinG is a large scaffolding protein (two splice variants of 270 kD and 480 kD have been reported at the AIS and nodes [15]) that participates in numerous protein–protein interactions, the following question arises: Why should Na^+^ channels and KCNQ2/3 K^+^ channels evolve nearly identical anchor motifs? One possible explanation is that structural constraints in ankyrinG limit the number of available binding sites for interacting proteins, resulting in Na^+^ and KCNQ2/3 K^+^ channels competing for the same binding site. In this scenario, neurons could use this competition to modulate and change the biophysical properties of their spike-generating machinery at the AIS in response to different signals. Intriguingly, recent evidence also indicates that sequences in and adjacent to the Na^+^ channel AIS anchor motif can be phosphorylated by the kinase CK2, thereby increasing a channel's affinity for ankyrinG by ∼1,000-fold [16]. It is interesting to speculate that given the central role of the AIS in AP initiation, additional levels of control may have evolved to permit dynamic and plastic regulation of AIS firing properties.

Besides initiating the AP, the AIS also functions to maintain neuronal polarity. Loss of ankyrinG completely disrupts neuronal polarity, leading to axons with the molecular characteristics of dendrites [5]. In the future, it will be interesting to determine key points in evolution that resulted in clearly defined neuronal polarity. One critical question will be whether clustering of Na^+^ channels was driven by neuronal polarity, or vice versa. These questions are appealing and compelling not only because they provide clues into the origins of ion channel clustering, but because they provide a window into the very origins of our own success.

## References

[1] Kole MH, Ilschner SU, Kampa BM, Williams SR, Ruben PC (2008). Action potential generation requires a high sodium channel density in the axon initial segment.. Nat Neurosci.

[2] Schwarz JR, Glassmeier G, Cooper EC, Kao TC, Nodera H (2006). KCNQ channels mediate IKs, a slow K+ current regulating excitability in the rat node of Ranvier.. J Physiol.

[3] Shah MM, Migliore M, Valencia I, Cooper EC, Brown DA (2008). Functional significance of axonal Kv7 channels in hippocampal pyramidal neurons.. Proc Natl Acad Sci U S A.

[4] Winckler B, Forscher P, Mellman I (1999). A diffusion barrier maintains distribution of membrane proteins in polarized neurons.. Nature.

[5] Hedstrom KL, Ogawa Y, Rasband MN (2008). AnkyrinG is required for maintenance of the axon initial segment and neuronal polarity.. J Cell Biol.

[6] Hedstrom KL, Xu X, Ogawa Y, Frischknecht R, Seidenbecher CI (2007). Neurofascin assembles a specialized extracellular matrix at the axon initial segment.. J Cell Biol.

[7] Zhou D, Lambert S, Malen PL, Carpenter S, Boland LM (1998). AnkyrinG is required for clustering of voltage-gated Na channels at axon initial segments and for normal action potential firing.. J Cell Biol.

[8] Dzhashiashvili Y, Zhang Y, Galinska J, Lam I, Grumet M (2007). Nodes of Ranvier and axon initial segments are ankyrin G-dependent domains that assemble by distinct mechanisms.. J Cell Biol.

[9] Garrido JJ, Giraud P, Carlier E, Fernandes F, Moussif A (2003). A targeting motif involved in sodium channel clustering at the axonal initial segment.. Science.

[10] Lemaillet G, Walker B, Lambert S (2003). Identification of a conserved ankyrin-binding motif in the family of sodium channel alpha subunits.. J Biol Chem.

[11] Pan Z, Kao T, Horvath Z, Lemos J, Sul J-Y (2006). A common ankyrin-G-based mechanism retains KCNQ and Nav channels at electrically active domains of the axon.. J Neurosci.

[12] Hill AS, Nishino A, Nakajo K, Zhang G, Fineman JR (2008). Ion channel clustering at the axon initial segment and node of Ranvier evolved sequentially in early chordates.. PLoS Genet.

[13] Teravainen H, Rvainen CM (1971). Fast and slow motorneurons to body muscle of the sea lamprey.. J Neurophysiol.

[14] Susuki K, Rasband MN (2008). Molecular mechanisms of node of Ranvier formation.. Curr Opin Cell Biol.

[15] Kordeli E, Lambert S, Bennett V (1995). AnkyrinG. A new ankyrin gene with neural-specific isoforms localized at the axonal initial segment and node of Ranvier.. J Biol Chem.

[16] Bréchet A, Fache MP, Brachet A, Ferracci G, Baude A (2008). Protein kinase CK2 contributes to the organization of sodium channels in axonal membranes by regulating their interaction with ankyrin G.. J Cell Biol.

